# Candidate Genes for Inherited Autism Susceptibility in the Lebanese Population

**DOI:** 10.1038/srep45336

**Published:** 2017-03-30

**Authors:** Silva Kourtian, Jihane Soueid, Nadine J. Makhoul, Dikran Richard Guisso, Maria Chahrour, Rose-Mary N. Boustany

**Affiliations:** 1 American University of Beirut Medical Center Special Kids Clinic, Neurogenetics Program and Division of Pediatric Neurology, Lebanon.; 2 Department of Biological and Environmental Sciences, Faculty of Science, Beirut Arab University, Lebanon; 3 Eugene McDermott Center for Human Growth and Development, Departments of Neuroscience and Psychiatry, University of Texas Southwestern Medical Center, Dallas, Texas, USA; 4 Biochemistry and Molecular Genetics, American university of Beirut, Lebanon

## Abstract

Autism spectrum disorder (ASD) is characterized by ritualistic-repetitive behaviors and impaired verbal/non-verbal communication. Many ASD susceptibility genes implicated in neuronal pathways/brain development have been identified. The Lebanese population is ideal for uncovering recessive genes because of shared ancestry and a high rate of consanguineous marriages. Aims here are to analyze for published ASD genes and uncover novel inherited ASD susceptibility genes specific to the Lebanese. We recruited 36 ASD families (ASD: 37, unaffected parents: 36, unaffected siblings: 33) and 100 unaffected Lebanese controls. Cytogenetics 2.7 M Microarrays/CytoScan™ HD arrays allowed mapping of homozygous regions of the genome. The *CNTNAP2* gene was screened by Sanger sequencing. Homozygosity mapping uncovered *DPP4, TRHR*, and *MLF1* as novel candidate susceptibility genes for ASD in the Lebanese. Sequencing of hot spot exons in *CNTNAP2* led to discovery of a 5 bp insertion in 23/37 ASD patients. This mutation was present in unaffected family members and unaffected Lebanese controls. Although a slight increase in number was observed in ASD patients and family members compared to controls, there were no significant differences in allele frequencies between affecteds and controls (C/TTCTG: γ^2^ value = 0.014; p = 0.904). The *CNTNAP2* polymorphism identified in this population, hence, is not linked to the ASD phenotype.

Autism spectrum disorder (ASD) is a neurodevelopmental condition characterized by repetitive/restrictive behaviors, problems in social interactions, and difficulties in communication. Even though the age of onset may be as early as 6 months, ASD is typically diagnosed after 18 months of age. Prevalence estimates of ASD in the general population have increased over the last 20 years. In 2010, 1 in 68 children in the USA was documented as affected, with 1 in 42 boys and 1 in 189 girls diagnosed with ASD. Small scale studies in Middle Eastern countries, including the gulf region and Saudi Arabia, found that in the latter 12.5% of children under the age of 18 years have ASD^1^. In Lebanon, the load of pediatric patients with ASD is increasing, and recently an incidence of 1 in 66 was reported in the greater Beirut and Mount Lebanon areas, with affected male to affected female ratios reported as 1:1.01^2^. In the past decade, the high concordance in monozygotic twins (>95%) compared to dizygotic twin pairs (22–65%)^3^ pointed to underlying genetic etiologies for ASD. Several factors had hindered identification of disease genes^4^,^5^,^6^,^7^ such as clinical variability, genetic heterogeneity, and contribution of environmental as well as epigenetic factors. Recurrent copy number variants (CNVs), however, have been identified, such as the maternally derived duplication of chromosome 15q11.2–13 identified in 0.5–3% of ASD cases^8^. Moreover, ASD phenotypes often occur in monogenic neurodevelopmental syndromes such as Tuberous Sclerosis, Fragile X, and Rett syndrome. These have autism as one of their main clinical features but only account for 2–5% of autism cases^9^,^10^,^11^. Under the new DSM-5 diagnostic criteria they have now been dropped from the umbrella of ASD. More recent whole exome sequencing studies, identified *de novo* mutations in up to 15% of ASD case^12^,^13^. Both CNVs and point mutations in genes such as *CNTNAP2, SHANK3, NLGN3, NLGN4*, and *NRXN1* have been reported in cases of ASD^14^,^15^,^16^.

The genetic makeup of the Lebanese population is unique due to significant shared ancestry, and DNA recombination events occurring over millennia, all these accompanied by high rates of consanguinity. The distinctive genetic fingerprint of the Lebanese population is well suited for comparative genomic hybridization (CGH) microarray analysis, homozygosity mapping, and CNV analysis for discovery of novel as well as previously described recessive disease-causing genes in ASD. Array CGH has improved the identification of putative ASD genes and increased molecular diagnosis in children with ASD to 25%. Demarcation of runs of homozygosity in family members allows immediate exclusion of thousands of genes as potentially causative. Homozygosity mapping in pedigrees with shared ancestry provides a fruitful methodology to identify whether genes considered under an autosomal recessive disease model can lead to ASD^17^,^18^. Genome-wide analysis locates genetic variations and identifies disease loci by genotyping parents, affected, and unaffected progeny. Disease loci appear in regions where only affected individuals are homozygous for the suspected culprit allele.

In the present study, microarray analysis was used to identify runs of homozygosity (ROH) unique to affected ASD patients. The occurrence of identical ROH regions in at least 5 out of 37 affected individuals shed light on novel ASD susceptibility candidate genes in these regions. One of the genes that were common to 9 ROH regions in our cohort is *CNTNAP2*. It encodes a neuronal cell adhesion molecule that interacts with Contactin 2 (Cntn2) at the juxtaparanodal region at the nodes of Ranvier. These are regularly spaced gaps between the myelin-producing Schwann cells in the peripheral nervous system (PNS)^19^,^20^. While previous investigations have focused on the role of *CNTNAP2* in PNS development, a recent report demonstrated that a homozygous *CNTNAP2* mutation in the Old Order Amish population results in intractable seizures, histologically confirmed cortical neuronal migration abnormalities, and ASD^21^. It is hypothesized that ASD may result from improper excitatory/inhibitory balance brought by the inability to form adequate synaptic connections^22^. We screened *CNTNAP2* for mutations in our cohort of ASD patients, their family members, and unaffected controls, and identified a novel polymorphism in the Lebanese population.

## Results

All affected patients satisfied DSM4/5 criteria and were evaluated by a pediatric neurologist, pediatric psychiatrist and psychologists. Family history and clinical assessment of the phenotypic features are summarized in Table 1). Microarray analysis and mapping of ROH were performed in each family. Assuming a model of autosomal recessive inheritance and high penetrance, only ROHs unique to the affected patient(s) were considered (Table 1).

Of these ROH, only those found in 5 or more out of the 37 affected children were analyzed, particularly if they were overlapping ROH in different patients/families (Table 2). We found one stretch of ROH common to 5 children with ASD, located on chromosome 8q23.1, two ROH common to 6 children with ASD, one located on chromosome 2p22.1 and the other located on chromosome 3q25.32, and one ROH common to 7 children with ASD located on chromosome 18q11.2. ASD-associated copy number variations spanning these ROH regions in part, or whole, of all 4 ROH regions listed below have been reported in individuals with autism in the Simons Foundation Autism Research Initiative (SFARI) gene database.

**Figure 1:**
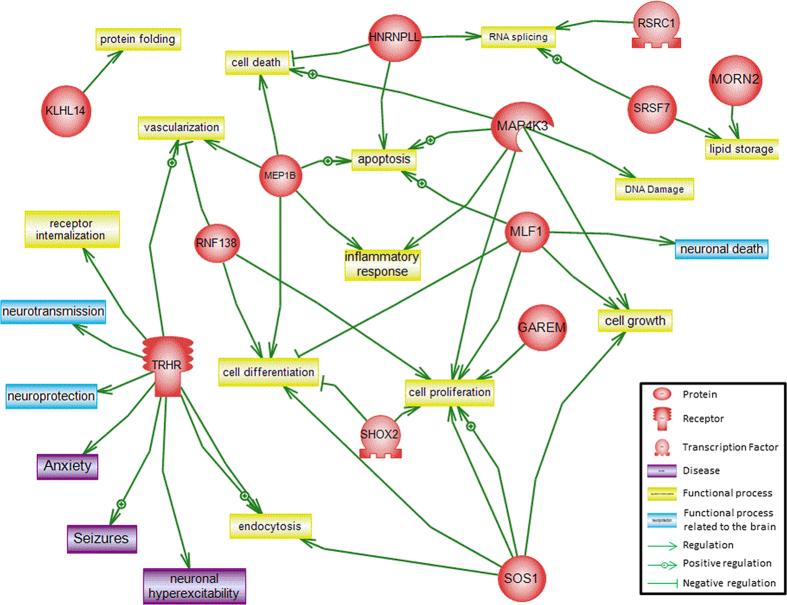
Biological network created using pathway analysis to identify biological functions and disorders associated with genes selected from ROH regions common to at least 5 ASD patients in an overlapping manner. Thirteen genes are depicted involved in direct interactions. Cellular processes are highlighted in yellow, cellular processes related to the brain are in blue, and disorders are in purple.

**Figure 2:**
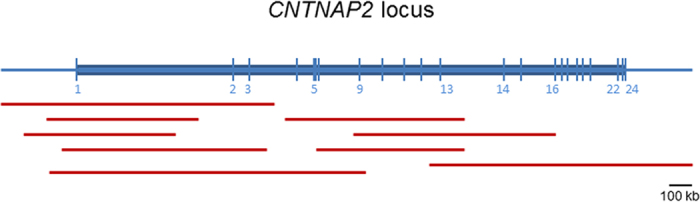
The human *CNTNAP2* locus at 7q35. Schematic indicating the 24 exons (blue bars) of *CNTNAP2*. Red lines indicate ROH regions found in 7 ASD patients. Note that there is no complete overlap of ROH regions between the 7 patients.

**Figure 3:**
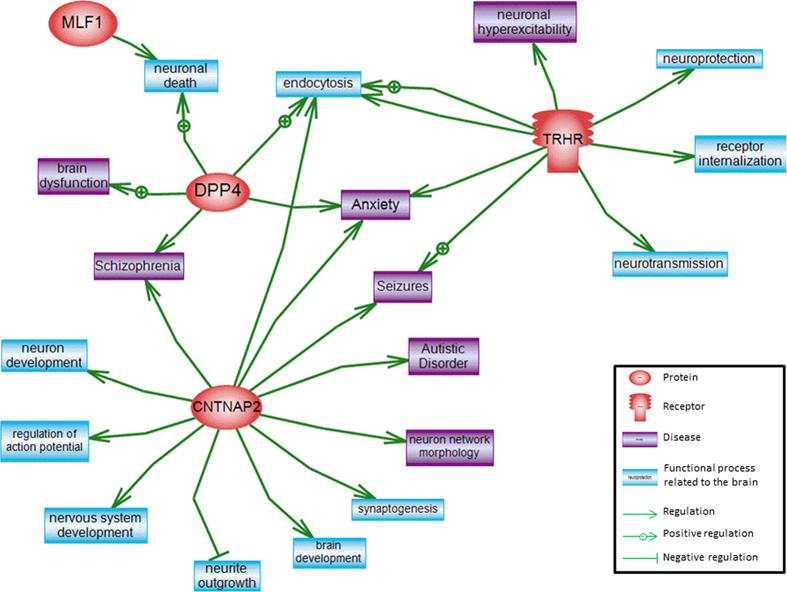
Biological network created using pathway analysis linking genes of interest *DPP4, TRHR, MLF1*, and *CNTNAP2* to cellular processes related to the brain (in blue) and neurological disorders (in purple).

Pathway analysis of the genes within these 4 ROH regions did not reveal relationships amongst those genes, but linked one of them (*TRHR*) to neurological diseases including seizures, anxiety, and neuronal hyperexcitability (Fig. 1). The genes were involved in different cell processes such as apoptosis, cell differentiation, cell proliferation, DNA damage, RNA splicing, inflammatory response, endocytosis, cell growth, vascularization, and lipid storage. Only *TRHR* and *MLF1* implicated cell processes related to the brain. In addition to these 2 genes, the analysis of ROH regions common to at least 4 ASD patients uncovered more genes (data not shown), one of them, *DPP4,* was of particular interest. We also identified additional nine ROH found in 7 ASD patients that were not completely overlapping, but they all occurred within one gene, *CNTNAP2* (Fig. 2). Network analysis of *TRHR, MLF1, DPP4*, and *CNTNAP2* showed relations of these genes to neuronal processes including endocytosis, neurite outgrowth, synaptic transmission, neuronal death as well as neuron development and neurite outgrowth, regulation of action potential and synaptic transmission, in addition to brain and neuron development (Fig. 3). Both the newly identified *DPP4*, previously linked to schizophrenia and brain dysfunction, and *TRHR*, previously linked to neuronal hyperexcitability, seizures, and anxiety may be linked to ASD in our cohort.

**Figure 4:**
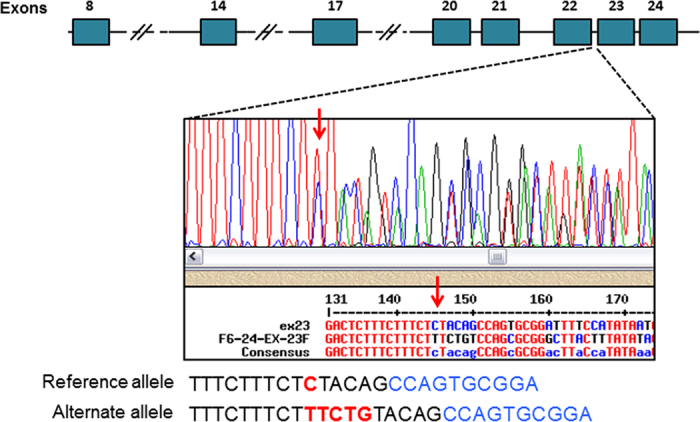
An insertion of 5 bp (chr7: 148,106,477 C > TTCTG) upstream of exon 23 (red arrow) of CNTNAP2. Representative Sanger sequence data depicting the 5 bp insertion. Intronic sequence is shown in black, coding sequence in blue, and the insertion in red.

*CNTNAP2* is the only gene to be previously reported in ASD. We performed mutational screening for *CNTNAP2* in our cohort, and only exons previously reported as harboring mutations were screened (exons 8, 9, 10, 11, 12, 13, 14, 17, 20, 21, 22, 23, and 24). A 5 bp insertion (Fig. 4, red arrow) was detected in the intron upstream of exon 23 of *CNTNAP2*. This mutation was found in 23 out of 37 unrelated ASD patients we screened. Eight ASD patients were homozygous for this insertion and fifteen ASD patients were heterozygous (Table 3).

Genotyping for *CNTNAP2* in our cohort of 106 total individuals consisting of ASD patients and their family members revealed 32 (30.2%) individuals with the NN genotype (homozygous for the reference allele), 44 (41.5%) individuals with the NV genotype, and 30 (28.3%) individuals with the VV genotype (homozygous for the alternate allele) (Table 3). Moreover, the screening of 100 unaffected control individuals with no family history of ASD uncovered 34 (34%) individuals with the NN genotype, 50 (50%) individuals with the NV genotype, and 16 (16%) individuals with the VV genotype (Table 3). There was no significant difference between ASD families and the control group for this polymorphism (γ^2^ value = 4.5335; p = 0.103). The alternate (V) allele frequencies in cases and controls were 0.49 and 0.41, respectively, however, this difference was not statistically significant (γ^2^ value = 0.014; p = 0.904). Furthermore, this particular variant (chr7: 148,106,477 C > TTCTG) was absent from 60,706 unrelated individuals in the Exome Aggregation Consortium (ExAC) server. Since the majority of ExAC data pertains to individuals of European ancestry, it is not surprising that our variant identified in a Lebanese cohort was absent from ExAC.

## Discussion

We identified three novel candidate ASD genes, *DPP4, THRH*, and *MLF1*, and network analysis provided arguments in favor of these genes. *DPP4* is directly related to anxiety and schizophrenia. It plays a role in endocytosis and neuronal death as well as brain dysfunction. Although *DPP4* had never been directly associated with autism, some findings link it to autistic features. Recent studies show that a decrease in the gene expression or activity of *DPP4* would result in possible neurological consequences and exacerbation of autism symptoms^23^. Moreover, a deletion encompassing *DPP4* among other genes was found in a patient presenting with hypotonia, delayed motor development, severe language impairment, and behavior consistent with ASD^24^. In another study, *DPP4* was linked to attention problems and aggressiveness. Increased activity of DPP4 leads to a reduced amount of oxytocin and vasopressin, with low levels of these neuropeptides leading to increased aggressiveness and negative social behavior^25^. In a rodent model, targeted inactivation of the *Dpp4* gene resulted in healthy knockout mice. *Dpp4*^−/−^ homozygous mutants showed hypoglycemia, hyperinsulinemia, and increased plasma glucagon-like peptide 1 in glucose tolerance tests^26^. Behavior of these mice by analyzing “behavioral despair” is reported^27^. This behavior consists of failure to escape from aversive stimuli such as anxiety, curiosity, and motor activity. This is similar behavior following chronic administration of antidepressants. Along with reduced depressive-like behavior, *Dpp4*^−/−^ mice show higher novelty-induced hyperactivity^27^. These reported findings impart a strong role for DPP4 expression in the regulation of mood.

The thyrotropin-releasing hormone receptors (TRHR) are found in the anterior pituitary and in neurons throughout the central nervous system^28^,^29^,^30^. The neuroanatomical location and neurochemical actions of TRH and its receptors suggest that it could be utilized as a therapeutic agent in neurological and psychiatric disorders. Several studies of TRH effect in patients with depression reported an improvement in anxiety as well as in depressive mood^31^,^32^,^33^. TRH may also protect against rather than induce seizures^34^,^35^. It also demonstrates neuroprotective effects^36^,^37^, and may be involved in chronic neuronal hyperexcitability associated with kindling^36^. More controversial reports have suggested that TRH administration may be of therapeutic benefit for patients afflicted with schizophrenia^38^,^39^. *Trhr1* knockout mice have central hypothyroidism and mild hyperglycemia but exhibit normal growth and development as well as normal body weight and food intake^40^. Behaviorally, *Trhr1*^−/−^ mice display increased anxiety and depression^41^.

Although Myelodysplasia/myeloid leukemia factor 1 gene (*MLF1*) did not show any relation to psychiatric disease, this gene has a role in the brain. Initially discovered to be involved in a chromosomal translocation associated with myelodysplastic syndrome and acute myeloid leukemia^42^, it has been determined that MLF1 has protective effects on neuronal cell death in drosophila^43^. In mice, *Mlf1*^−/−^ embryos failed to develop beyond embryonic day 6.5^44^.

The CNTNAP2 protein, a member of the neurexin superfamily, has been repeatedly associated with a wide spectrum of neuropsychiatric disorders, such as developmental language disorders^45^, autism^19^,^46^,^47^, epilepsy^21^, and schizophrenia^48^. This cell adhesion molecule is crucial for the maintenance of functional synapses^49^,^50^. A mouse knockout model created in 2007 shows features of autism and seizures^51^. It has been suggested that *CNTNAP2* common variants may represent susceptibility factors for other language-related deficits such as specific language impairments, in addition to autism^52^. Heterozygous mutations in *CNTNAP2* are frequently associated with susceptibility to autism. *CNTNAP2* was also identified as an ASD causative gene due to its association to semantic-pragmatic skills and social inhibition^53^. A deletion in *CNTNAP2* leads to delayed myelination in the brain with abnormal T2 hyperintensities and causes loss of white matter volume^54^. In addition, a homozygous frameshift mutation in *CNTNAP2* was discovered in an Amish family with syndromic ASD (Cortical dysplasia focal epilepsy syndrome), a neuron migration disorder with epilepsy, language regression, hyperactivity, intellectual disability, and ASD^55^. In our study, *CNTNAP2* encompasses nine additional ROH found in 7 ASD patients. Mutational screening of all ASD patients, however, did not reveal any previously reported mutation. We did observe a novel 5 bp insertion in the intron upstream of exon 23. Although we observed the alternate homozygous genotype more frequently in the group of affected individuals and their family members compared to the unaffected control group (28.3% versus 16%, respectively, or a difference of 12.3%), the difference in allele frequency was not statistically significant (p > 0.05) between the two groups. Results, therefore, do not support this insertion as a high-risk genetic variant for ASD but as a benign polymorphism commonly found in the Lebanese population.

## Conclusion

This study highlights the effectiveness of microarray technology and homozygosity mapping in the search for ASD susceptibility genes in the Lebanese population. We propose in this study that *DPP4, TRHR*, and *MLF1* are 3 novel candidate genes for ASD. Mutational screening of *CNTNAP2*, which is classified as a strong autism risk gene, uncovered a novel *CNTNAP2* 5 bp intronic insertion upstream of exon 23 in Lebanese ASD cases and unaffected controls. Yet, one cannot exclude that this novel *CNTNAP2* polymorphism we uncovered may contribute to the ASD phenotype, depending on a second hit in the genome and other modifying factors.

## Methods

### Patient Samples

Blood was collected from 36 families, with 37 ASD patients (35 males and 2 females), who fulfilled Diagnostic and Statistical Manual of Mental Disorders-4/5 criteria for autism (Table 1). A protocol was developed with consent forms that were approved by the Institutional Review Board (IRB) of the American University of Beirut (IRB protocol number: BIOCH.RB.06 Protocol Name: Autism susceptibility genes in the Lebanese population). All experimental protocols were carried out in accordance with the relevant guidelines and approved by the relevant committees. Informed consent was obtained from the parents for themselves, siblings if above the age of twelve, and on behalf of children with ASD and those below 12 years of age from the parents by the investigators who are all CITI certified. In addition, a control cohort of 100 unaffected participants was recruited. DNA samples were extracted from peripheral blood using QIAamp^®^ blood midi kit (Qiagen, Inc., Valencia, CA).

### Microarray Analysis

Affymetrix Cytogenetics Whole-Genome 2.7 M Microarrays and CytoScan**™** HD arrays carry 2,700,000 unique, non-polymorphic probes covering the whole genome for detection of 35 kb or higher copy number variants as well as runs of homozygosity (ROH). Analysis was performed according to manufacturer protocols (Affymetrix Inc., Santa Clara, CA). Briefly, 3 μl of genomic DNA (33 ng/μl) are denatured and neutralized, and then amplified by PCR. The PCR products are then purified using magnetic beads (Beckman Coulter, Beverly, MA). The purified PCR products are fragmented and end-labeled with biotin. The fragmented, labeled PCR products are then hybridized overnight to the arrays. The arrays are washed and stained using the GeneChip^®^ Fluidics Station 450, and DAT images are acquired using the GeneChip^®^ Scanner 3000 (Affymetrix Inc.). Microarray data are available in the ArrayExpress database (www.ebi.ac.uk/arrayexpress) under accession number E-MTAB-4963. Data analysis is performed using Affymetrix Chromosome Analysis Suite (CHAS). Pathway Studio software version 9.0 (Ariadne Genomics, Rockville, Md., USA) was used to identify pathways and biological processes common to genes within ROH regions and place them in networks related to neuronal disease or autism.

### PCR and Sanger Sequencing

Primer pairs listed in Table 4 were used to amplify coding exons of *CNTNAP2*, including intronic flanking regions, from genomic DNA with a standard polymerase chain reaction (PCR) over 35 cycles with a 56.7 °C annealing temperature. PCR reactions were performed using 100 ng of DNA, 50 ng of each primer, 1X IQ Phusion mix (Finnzymes^®^) containing buffer, dNTPs, and Taq polymerase. PCR products were purified on 1% agarose gel using the GE Healthcare^®^ DNA purification kit. DNA was then screened for mutations by unidirectional direct sequencing using the BigDye^®^ Terminator v1.1 Cycle Sequencing Kit (Applied Biosystems, Foster City, CA). Mutations were confirmed with an independent PCR and bidirectional sequencing.

## Additional Information

**How to cite this article**: Kourtian, S. *et al*. Candidate Genes for Inherited Autism Susceptibility in the Lebanese Population. *Sci. Rep.* **7**, 45336; doi: 10.1038/srep45336 (2017).

**Publisher's note:** Springer Nature remains neutral with regard to jurisdictional claims in published maps and institutional affiliations.

## Figures and Tables

**Table 1:** Characteristics of patients with autism included in the study.

Patient ID	Age	Gender	Total Number of ROH in the genome	Locus of overlapping ROH regions	Satisfied DSM4/5 criteria	Family history	Additional notable clinical features
F1-001	2y	Male	65	2p22.1	+	Negative for psychiatric/mental disorder	Tip-toe walking, non-verbal
F2-005	14y	Male	29		+	Mental retardation in mother’s uncle.	Non-verbal, ECAR-T score** = **61
F3-010	23y	Male	68	2p22.1 18q12.1	+	Negative for psychiatric/mental disorder	N.A.
F4-015	17y	Male	44	8q23.1 3q25.32 18q12.1	+	Negative for psychiatric/mental disorder	ECAR-T score = 31
F5-020	5y	Male	50		+	Depression in maternal grand-father	N.A.
F6-024	10y	Male	41	2p22.1 8q23.1 3q25.32	+	Negative for psychiatric/mental disorder	N.A.
F7-029	21y	Male	35	8q23.1	+	Asperger’s case in maternal family	N.A.
F8-034	14y	Male	77		+	Negative for psychiatric/mental disorder	N.A.
F9-038	10y	Male	113	8q23.1	+	Negative for psychiatric/mental disorder	N.A.
F11-048	7y	Male	36		+	Negative for psychiatric/mental disorder	Verbal, talked at 2y
F15-070	6y	Male	11		+	Negative for psychiatric/mental disorder	Agoraphobia
F17-080	2y	Male	59		+	Father has autistic features	Hyperactive, speech delay
F18-083	13y	Male	45	2p22.1	+	Negative for psychiatric disorder. Mental retardation in a family member	Speech delay
F19-088	4y	Male	11	2p22.1	+	Negative for psychiatric disorder. Mental retardation in a family member	High IQ, speech regression at 2.5y, aggressive, mild ASD
F19-089	3y	Male	11	2p22.1	+	Negative for psychiatric/mental disorder	Severe autistic features, echolalia, speech regression at 2y, mental retardation
F20-093	3y	Male	49	3q25.32 18q12.1	+	Negative for psychiatric/mental disorder	Speech regression at 2y, stereotypies, little improvement with therapies
F22-101	5y	Male	47	18q12.1	+	Mental retardation in paternal cousin	Congenital cataract, Hyperactivity
F24-108	8y	Male	5		+	Negative for psychiatric/mental disorder	Speech regression at 2y
F27-122	8y	Male	35	3q25.32	+	Negative for psychiatric/mental disorder	N.A.
F28-127	9y	Male	29	3q25.32 18q12.1	+	Negative for psychiatric/mental disorder	N.A.
F29-133	8y	Male	41	2p22.1	+	Negative for psychiatric/mental disorder	N.A.
F31-145	3y	Male	47		+	Negative for psychiatric/mental disorder	Hyperactive, stereotypies, speech delay
F32-148	8y	Male	38		+	Negative for psychiatric/mental disorder	Speech delay at 3.5y, significant improvement with therapies
F33-152	2y	Male	31		+	Negative for psychiatric/mental disorder	Non-verbal, tip toe walking
F34-158	4y	Male	10		+	Negative for psychiatric/mental disorder	No stereotypical movements, aggressive, small improvement with therapies
F35-164	16y	Female	32	18q12.1	+	Negative for psychiatric/mental disorder	Non-verbal, hyperactive
F36-169	4y	Male	61	3q25.32 18q12.1	+	Family history of autism	Non-verbal.
F37-172	8y	Male	44		+	Negative for psychiatric/mental disorder	Walked at 2.5y, non-verbal, stereotypies, Krabbe disease
F38-176	10y	Male	37		+	Negative for psychiatric/mental disorder	Delayed speech, walked at 1y 4 m, aggressive, improved with therapies
F39-180	8y	Male	18		+	Negative for psychiatric/mental disorder	Walked at 1.6y, few words, epilepsy
F40-186	5y	Male	57	8q23.1	+	Mother had intra-partum depression	Speech regression, rigid behavior, stereotypies
F41-190	15y	Male	37		+	Father has depression. Complications during labor and delivery	Talked at 1y5m, stereotypies, rigid behavior, echolalia
F42-194	7y	Male	25		+	Mother has depression	Stereotypies, non-verbal, delayed walking
F43-197	N.A	Male	26		+	Negative for psychiatric/mental disorder	Feeding difficulties in early childhood, drooling, flapping tip -toe walking, Non-verbal, improvement with therapies
F44-201	3y	Male	20		+	Negative for psychiatric/mental disorder	Speech delay, stereotypies
F45-205	12y	Male	21		+	Asperger’s case on the maternal side	Hyperactivity, non-verbal, improvement with therapies
F46-211	N.A	Female	51		+	Negative for psychiatric/mental disorder	Regression at 2y, tip-toe walking, stereotypies, non-verbal

Total number of ROH in the genome are only ROHs unique to the affected patient(s) and not present in the parents (column 4). Locus of overlapping ROH regions found at least in 5 ASD patients and described in table 2 (see column 5). N.A: Data not available.

**Table 2:** ROH regions found in at least 5 ASD patients.

Locus	Overlapping ROH coordinates	Length (Kb)	Gene content	ASD patients
8q23.1	110051909–110185293	820	*TRHR*	F4-015
				F6-024
				F7-029
				F9-038
				F40-186
3q25.32	157813799–158331136	517	*MLF1 RSRC1 SHOX2*	F4-015
				F6-024
				F20-093
				F27-122
				F28-127
				F36-169
2p22.1	38790327–39664219	873	*SOS1 MAP4K3 CDKL4 HNRPLL SRSF7 DHX57 MORN2 ARHGEF33*	F1-001
				F3-010
				F6-024
				F18-083
				F19-88
				F19-89
				F29-133
18q12.1	29687013–30278521	591	*RNF138 WBP11P1 FAM59A KLHL14 MEP1B*	F3-010
				F4-015
				F20-093
				F22-101
				F28-127
				F35-164
				F36-169

ROH chromosomal locations and coordinates for the overlapping regions are shown (human genome build hg19). Genes within the ROH are listed. ASD patients with the corresponding ROH are indicated (out of the 37 ASD patients in our cohort).

**Table 3:** Genotype and allelic distribution of *CNTNAP2* variant in ASD patients, their family members, and unaffected controls.

	ASD patients (n = 37)	Parents (n = 36)	Unaffected siblings (n = 33)	Patients and family members (n = 106)	Controls (n = 100)
Genotype					
NN	14 (38%)	7 (19.4%)	11 (33.3%)	32 (30.2%)	34 (34%)
NV	15 (40%)	19 (52.8%)	10 (30.3%)	44 (41.5%)	50 (50%)
VV	8 (22%)	10 (27.8%)	12 (36.4%)	30 (28.3%)	16 (16%)
N allele frequency	0.58	0.46	0.48	0.51	0.59
V allele frequency	0.42	0.54	0.52	0.49	0.41

N: reference allele; V: alternate allele with the TTCTG insertion at intron/exon junction of exon 23.

**Table 4:** Primer sequences for amplification and sequencing of *CNTNAP2* coding exons.

Exon	Primer sequence	Amplicon size (bp)
CNTNAP2_ex8F	tcactgaatccatgctctgc	524
CNTNAP2_ex8R	aaaacctaatcctgagcgtgtaac	
CNTNAP2_ex14F	agagtattcctggggaagtgg	440
CNTNAP2_ex14R	ttgtcgcactgacctctttct	
CNTNAP2_ex17F	tcgacctttgtaggacgtgac	479
CNTNAP2_ex17R	ggccaacacctttacttttgg	
CNTNAP2_ex20F	agcaggaattgaggggatgt	350
CNTNAP2_ex20R	ttatgcacttgtaggagaaagtgt	
CNTNAP2_ex21F	gaaaaccagggttcaaagagtg	314
CNTNAP2_ex21R	aagatattcgtgactggccc	
CNTNAP2_ex22F	gctttggacacaagcattca	462
CNTNAP2_ex22R	acgttcctttgccctttctt	
CNTNAP2_ex23F	gttgtgattcttgtgggagaca	366
CNTNAP2_ex23R	cagcaaaatgaataatgtaaaaacc	
CNTNAP2_ex24F	gagagggctgtgtctgacg	437
CNTNAP2_ex24R	atattccattgcctgcctcc	
